# A multitrait genetic study of hemostatic factors and hemorrhagic transformation after stroke treatment

**DOI:** 10.1016/j.jtha.2023.11.027

**Published:** 2023-12-15

**Authors:** Cristina Gallego-Fabrega, Gerard Temprano-Sagrera, Jara Cárcel-Márquez, Elena Muiño, Natalia Cullell, Miquel Lledós, Laia Llucià-Carol, Jesús M. Martin-Campos, Tomás Sobrino, José Castillo, Mònica Millán, Lucía Muñoz-Narbona, Elena López-Cancio, Marc Ribó, Jose Alvarez-Sabin, Jordi Jiménez-Conde, Jaume Roquer, Silvia Tur, Victor Obach, Juan F. Arenillas, Tomas Segura, Gemma Serrano-Heras, Joan Marti-Fabregas, Marimar Freijo-Guerrero, Francisco Moniche, Maria del Mar Castellanos, Alanna C. Morrison, Nicholas L. Smith, Paul S. de Vries, Israel Fernández-Cadenas, Maria Sabater-Lleal, Cohorts for Heart and Aging Research in Genomic Epidemiology Consortium, Spanish Stroke Genetic Consortium

**Affiliations:** 1 Stroke Pharmacogenomics and Genetics Group, Institut de Recerca Sant Pau (IR SANT PAU), Barcelona, Spain; 2 Genomics of Complex Disease Group, Institut de Recerca Sant Pau (IR SANT PAU), Barcelona, Spain; 3 Neurology Unit, Hospital Universitari MútuaTerrassa, Terrassa, Spain; 4 Clinical Neurosciences Research Laboratories, Health Research Institute of Santiago de Compostela (IDIS), Santiago de Compostela, Spain; 5 Department of Neurology, Hospital Clínico Universitario de Santiago (CHUS), Santiago de Compostela, Spain; 6 Department of Neuroscience, Hospital Universitario Hermanos Trias y Pujol (HUGTP), Badalona, Spain; 7 Stroke Unit, Neurology Department, Hospital Universitario Central de Asturias (HUCA), Instituto de Investigación Sanitaria del Principado de Asturias (ISPA), Oviedo, Spain; 8 Stroke Unit, Hospital Universitario Valle de Hebrón (HUVH), Barcelona, Spain; 9 Department of Neurology, Hospital Universitario Valle de Hebrón (HUVH), Universitat Autònoma de Barcelona (UAB), Barcelona, Spain; 10 Department of Neurology, Neurovascular Research Group, Instituto de investigaciones médicas Hospital del Mar (IMIM) Hospital del Mar, Barcelona, Spain; 11 Department of Neurology, Hospital Universitario Son Espases (HUSE), Mallorca, Spain; 12 Department of Neurology, Hospital Clínic i Provincial de Barcelona, Barcelona, Spain; 13 Department of Neurology, Hospital Clínico Universitario, University of Valladolid, Valladolid, Spain; 14 Department of Neurology, Complejo Hospitalario Universitario de Albacete (CHUA), Universidad de Castilla-La Mancha (UCLM), Albacete, Spain; 15 Research Unit, Complejo Hospital Universitario de Albacete (CHUA), Albacete, Spain; 16 Department of Neurology, Hospital de la Santa Creu i Sant Pau, IIB-Sant Pau, Barcelona, Spain; 17 Neurovascular Unit, Biocruces Bizkaia Health Research Institute, Bilbao, Spain; 18 Department of Neurology, Hospital Universitario Virgen del Rocio, Instituto de Biomedicina de Sevilla (IBIS), Seville, Spain; 19 Department of Neurology, Hospital Universitario de A Coruña (CHUAC), Biomedical Research Institute, A Coruña, Spain; 20 Human Genetics Center, Department of Epidemiology, Human Genetics, and Environmental Sciences, School of Public Health, the University of Texas Health Science Center at Houston, Houston, Texas, USA; 21 Department of Epidemiology, University of Washington, Seattle, Washington, USA; 22 Kaiser Permanente Washington Health Research Institute, Kaiser Permanente Washington, Seattle, Washington, USA; 23 Department of Veterans Affairs Office of Research and Development, Seattle Epidemiologic Research and Information Center, Seattle, Washington, USA; 24 Cardiovascular Medicine Unit, Department of Medicine, Karolinska Institutet, Stockholm, Sweden

**Keywords:** fibrinogen, hemorrhagic transformation, hemostatic factors, r-tPA treatment, von Willebrand factor

## Abstract

**Background:**

Thrombolytic recombinant tissue plasminogen activator (r-tPA) treatment is the only pharmacologic intervention available in the ischemic stroke acute phase. This treatment is associated with an increased risk of intracerebral hemorrhages, known as hemorrhagic transformations (HTs), which worsen the patient’s prognosis. Objectives: To investigate the association between genetically determined natural hemostatic factors’ levels and increased risk of HT after r-tPA treatment.

**Methods:**

Using data from genome-wide association studies on the risk of HT after r-tPA treatment and data on 7 hemostatic factors (factor [F]VII, FVIII, von Willebrand factor [VWF], FXI, fibrinogen, plasminogen activator inhibitor-1, and tissue plasminogen activator), we performed local and global genetic correlation estimation multitrait analyses and colocalization and 2-sample Mendelian randomization analyses between hemostatic factors and HT.

**Results:**

Local correlations identified a genomic region on chromosome 16 with shared covariance: fibrinogen-HT, *P* = 2.45 × 10^−11^. Multitrait analysis between fibrinogen-HT revealed 3 loci that simultaneously regulate circulating levels of fibrinogen and risk of HT: rs56026866 (*PLXND1*), *P* = 8.80 × 10^−10^; rs1421067 (*CHD9*), *P* = 1.81 × 10^−14^; and rs34780449, near *ROBO1* gene, *P* = 1.64 × 10^−8^. Multitrait analysis between VWF-HT showed a novel common association regulating VWF and risk of HT after r-tPA at rs10942300 (*ZNF366*), *P* = 1.81 × 10^−14^. Mendelian randomization analysis did not find significant causal associations, although a nominal association was observed for FXI-HT (inverse-variance weighted estimate [SE], 0.07 [−0.29 to 0.00]; odds ratio, 0.87; 95% CI, 0.75–1.00; raw *P* = .05).

**Conclusion:**

We identified 4 shared loci between hemostatic factors and HT after r-tPA treatment, suggesting common regulatory mechanisms between fibrinogen and VWF levels and HT. Further research to determine a possible mediating effect of fibrinogen on HT risk is needed.

## Graphical Abstract

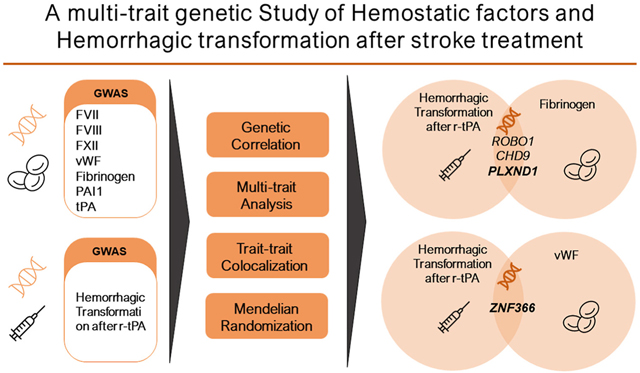

## 1 | INTRODUCTION

The initiation of treatment within the first hours after suffering from an acute ischemic stroke (IS) is crucial in producing better health outcomes [1]. Currently, the only available treatments in the acute phase are reperfusion therapies, such as thrombolysis and mechanical thrombectomy. Regardless of the benefits of thrombolytic recombinant tissue plasminogen activator (r-tPA) treatment, this is only offered to a low percentage of patients (15.6%) [2] due to a narrow therapeutic window [3] and potential severe side effects [3–5]. The most serious side effect is hemorrhagic transformation (HT), which involves disruption of the blood-brain barrier and extravasation of blood into the brain tissue, increasing the risk of mortality and worsening stroke outcomes. HT is influenced by a wide range of factors, including blood coagulation and fibrinolysis. According to the European Cooperative Acute Stroke Study, HT can be classified as hemorrhagic infarction or parenchymal hematoma (PH) [6].

Thrombolytic treatment presents a 6- to 7-fold increased risk of intracerebral hemorrhage [7]. Patients who develop PH have been shown to present a worse outcome and an increased risk of 3-month mortality [8]. Despite this, the most recent guidelines recommend administrating r-tPA to all eligible patients, even those undergoing mechanical thrombectomy [9], or elderly patients [10], and clinical trials have assessed the viability of extending the time window to thrombolysis therapy [11], which requires increased knowledge of the putative adverse effects of this treatment.

Although r-tPA has been used for more than 20 years, the biological processes associated with the risk of HT are largely unknown. To date, a few biomarkers have been studied to predict the risk of HT after r-tPA treatment, including blood levels of circulating proteins, such as matrix metalloproteinases, cellular fibronectin, or vascular adhesion protein-1 [2,12–15]. Additionally, some genetic variants (rs669, rs1801020, rs79770152, and rs76484331) have been associated with the response after r-tPA treatment [2,16,17]. A few studies have investigated the relationship between levels of various hemostatic factors and HT after r-tPA treatment [18–21]. Monitorization of the plasmin inhibitor complex, von Willebrand factor (VWF) levels, or fibrin/fibrinogen degradation products after r-tPA administration suggested that a test combining levels of these markers could aid in predicting intracerebral hemorrhages [18]. Intravenous thrombolysis using r-tPA results in increased perfusion of microvessels and decreased infarct size, likely due to depletion of plasma fibrinogen [19]. Additionally, recent clinical and experimental evidence suggests that ADAMTS-13 and VWF may be promising targets for thrombolysis of intracranial thrombi [20,21].

Genetically determined levels of hemostatic factors have been studied in relation to other vascular and cerebrovascular conditions. A Mendelian randomization (MR) study identified that factor F(XI) may be a viable target for reducing the risk of the cardioembolic subtype of IS [22]. Another MR study observed a causal relationship between FVIII and VWF and peripheral artery disease [23]. The development of specialized algorithms and statistical methods has allowed us to leverage existing summary statistics data from genome-wide association studies (GWASs) to identify new gene-phenotype associations. Using GWAS data from 2 different phenotypes, we can identify the proportion of variance that the 2 traits share, estimating the level of pleiotropy or causal overlap using global or local genetic correlations algorithms (linkage disequilibrium score regression [LDSC] [24] and SUPERGNOVA [25]). Multitrait analysis [26,27] is similar to meta-analysis in which 2 GWAS datasets from related phenotypes are combined to boost the power and aid the identification of new shared loci among them. Finally, 2-sample MR [28] is a statistical method that capitalizes on the random distribution of genetic variants that occurs at conception to avoid confounding and investigate causal relationships between an exposure (trait 1) and an outcome (trait 2).

We hypothesized that endogenous modifications in hemostatic factors that might alter the balance between thrombosis and bleeding might affect HT risk. With this study, we aimed to shed light on the relationship between several hemostatic factors’ genetically determined plasma levels and the risk of HT after tissue plasminogen activator (tPA) treatment by leveraging computational and statistical approaches that use existing GWAS data.

## 2 | METHODS

### 2.1 | Study design

**FIGURE 1:**
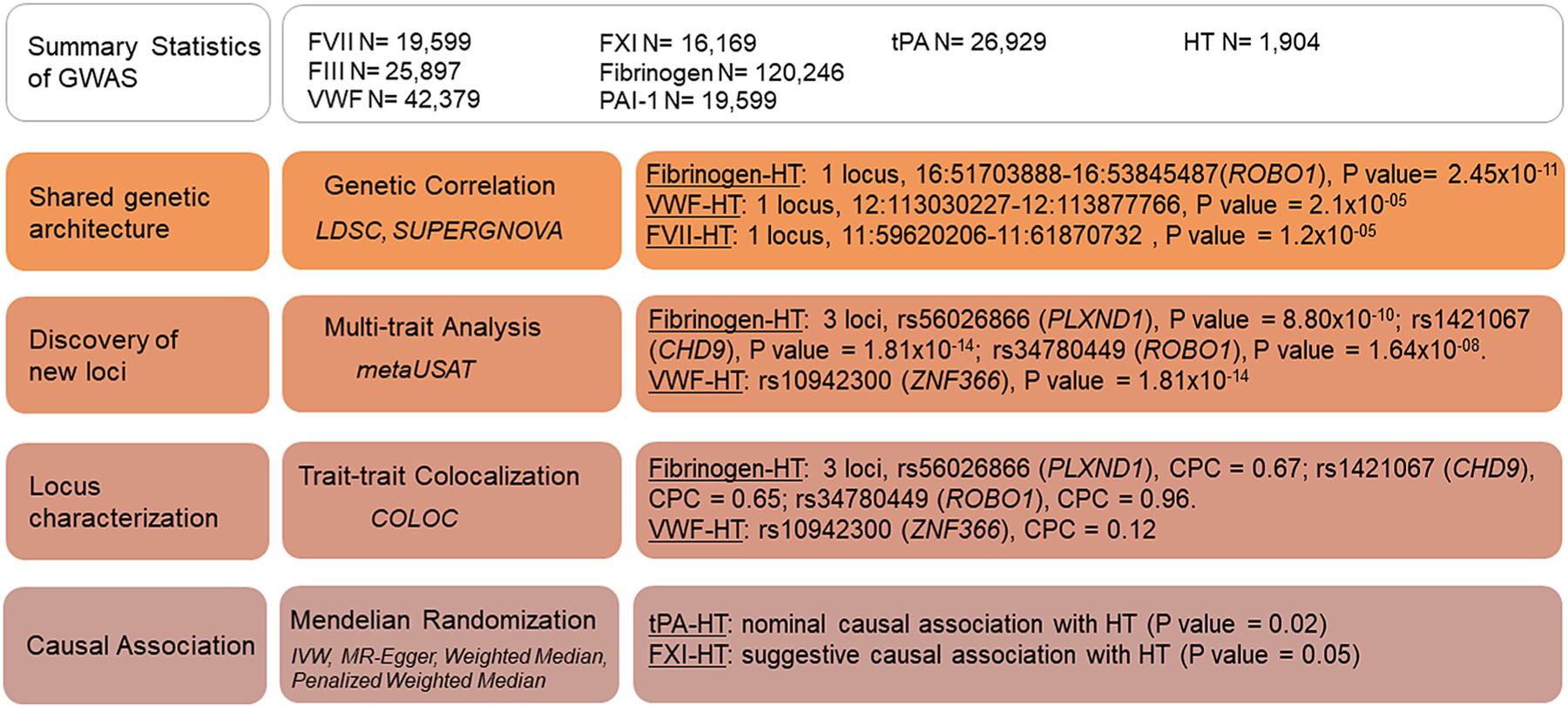
Graphic representation of the study design. CPC, conditional probability of colocalization; FVII, factor VII; FVIII, factor VIII; FXI, factor XI; GWAS, genome-wide association study; HT, hemorrhagic transformation; IVW, inverse-variance weighted; LDSC, linkage disequilibrium score regression; MR, Mendelian randomization; PAI-1, plasminogen activator inhibitor-1; tPA, tissue plasminogen activator; VWF, von Willebrand factor.

To evaluate the relationship between hemostatic factors and HT after r-tPA treatment, we used several approaches, including first, estimating global and local genetic correlations between each hemostatic factor and HT after r-tPA treatment using LDSC [24] and SUPERGNOVA [25], respectively, to estimate shared genetic heritability between traits. Since there were local points of genetic correlation, we then performed multitrait analyses between the 7 hemostatic factors and HT to identify loci that simultaneously regulate both phenotypes [26]. Finally, to discern if the observed relationships between hemostatic factors and HT were causal, we performed 2-sample MR [28]. Figure 1 contains a graphic representation of the study design.

### 2.2 | GWAS data sources

We used GWAS summary statistics investigating plasma levels or activity of 7 hemostatic factors (VWF [29], FXI [30], fibrinogen [31], plasminogen activator inhibitor-1 [PAI-1] [32], tPA [33], FVII [34], and FVIII [29]) and from a study reporting on HT after r-tPA treatment in the acute phase of an IS [17]. Characteristics and sample sizes of the GWAS datasets are listed in Table 1 [35]. In short, we used summary-level data from GWAS of hemostatic factors plasma levels from the Cohorts for Heart and Aging Research in Genomic Epidemiology Consortium [36,37]. For the present study, only data from European-ancestry individuals were used.

We also used summary-level data from the Genetic Study in Ischemic Stroke Patients treated with r-tPA (GenoTPA) [17] (Table 1). GenoTPA is a multicenter GWAS of European-ancestry patients with IS admitted to the emergency room at Spanish hospitals and treated with r-tPA alone (*N* = 2045). The study aimed at exploring genetic differences between patients presenting a PH after r-tPA treatment (*N* = 141; PH-1 = 69, PH-2 = 72) vs those without HT (non-HT) (*N* = 1904) [17]. Detailed information about the GenoTPA cohort and GWAS analysis [17] can be found in the Supplementary Methods and Supplementary Table S1.

### 2.3 | Standard protocol approvals and patient consent

All selected GWAS received ethical approval from their local committees. All patients included in the studies have provided informed consent for their participation.

### 2.4 | Genetic correlation and heritability estimates

LDSC [24] was used to estimate global genetic correlations between each pair of hemostatic factors’ plasma or activity levels and the hemorrhagic outcome after r-tPA treatment in patients with IS. Additionally, SUPERGNOVA [25] was used to calculate local genetic correlations between each pair of hemostatic factors’ plasma or activity levels and HT [25]. We used the genome partitions obtained with LDetect [38] from the 1000 Genomes Project [39], from European ancestry, to define the regions. We applied a Bonferroni correction to each individual pairing analysis to establish statistical significance (*P* < 3 × 10^−6^). Further details can be found in the Supplementary Methods.

### 2.5 | Multitrait analyses

Multitrait analyses were performed between each pair of the 7 hemostatic factors (FVII, FVIII, VWF, FXI, fibrinogen, PAI-1, and tPA) and the HT outcome after r-tPA treatment using the metaUSAT R package (v1.17) (R Core Team [2022]) [26].

To detect loci that regulate both the levels of a particular hemostatic factor and the risk of HT after r-tPA treatment, we selected loci with a lead variant with a *P* value <5 × 10^−8^ in the multiphenotype analysis, which was at least an order of magnitude smaller than the lowest *P* value in the individual phenotypes, and with a *P* value <5 × 10^−3^ for both individual phenotypes [40,41].

To define a locus, we selected variants that were in a genomic region ±500 kb around the lead variant or linkage disequilibrium r^2^ > 0.2 with the lead variant.

### 2.6 | Trait-trait colocalization

Genomic regions with significant correlations and significant common loci identified in the multitrait analyses were submitted to a colocalization analysis using the COLOC R package (v5) [42] to look for evidence of common genetic variants regulating both the expression of the hemostatic factor and the risk of HT after r-tPA treatment. We considered conditional probabilities of colocalization (CPCs) ≥ 0.8, defined as the probability that a common regulatory variant exists, assuming the existence of a signal in both traits (posterior probability of hypothesis [PPH] 4 ÷ [PPH3 + PPH4]), as significant colocalizations [43]. Further details on the calculation of conditional probabilities are shown in the Supplementary Methods.

### 2.7 | MR

#### 2.7.1 | Genetic instruments selection

We initially selected genetic variants that reached statistical significance (*P* < 5 × 10^−8^) in each GWAS for the 7 hemostatic factors. Variants were then pruned using an r^2^ cutoff of 0.05 and a 1-Mb window based on the 1000 Genome Project European ancestry panel for linkage disequilibrium reference [44]. Independent variants with the lowest *P* values in each window and present in the summary statistics of HT after r-tPA treatment were kept as instrumental variables for further MR analysis (Supplementary Tables S2 and S3).

#### 2.7.2 | Estimating causal effects

Two-sample MR was performed using the R package “TwoSampleMR” (v0.5.5) [45] using each hemostatic factor as exposure to test its potential causal effect on HT after r-tPA treatment. We considered the inverse-variance weighted meta-analysis method as the main MR method to combine the effect estimates of the variants associated with each hemostatic factor and required an adjusted q value of <.05. Further details on sensitivity analyses and power calculations are found in Supplementary Table S4 and in the Supplementary Methods.

All values were normalized, and results are expressed as odds ratios (ORs) in outcome risk per every SD change of the hemostatic factor.

### 2.8 | Reporting guidelines

A completed copy of the Strengthening the Reporting of Observational Studies in Epidemiology (STROBE) Statement: guidelines for reporting observational studies and its extension for MR analysis STROBE-MR are provided as Supplementary material.

## 3 | RESULTS

### 3.1 | Genetic correlation and heritability estimates

Estimates of global genetic correlations between FVII, FVIII, VWF, FXI, fibrinogen, PAI-1, and tPA with HT after r-tPA treatment are shown in Supplementary Table S5. Overall, we did not find significant global genetic correlations between hemostatic factors and HT after r-tPA treatment. Local correlations analyses identified 1 genomic region with local covariance between fibrinogen and HT on chromosome 16 (significant local covariance *P* = 2.45 × 10^−11^) after applying multiple comparisons for all phenotypes (*P* < 3 × 10^−6^) and 2 more suggestive regions after applying multiple comparisons correction within each phenotype (*P* < 2.2 × 10^−5^) (Table 2 and Supplementary Table S6).

### 3.2 | Multitrait analyses

We detected a total of 99 significant loci (*P* < 5 × 10^−8^) (Supplementary Table S7) across all multitrait analyses. Specifically, we detected significantly associated loci for all 7 analysis pairings: FVII-HT: 12 loci; FVIII-HT: 10 loci; VWF-HT: 18 loci; FXI-HT: 5 loci; fibrinogen-HT: 48 loci; PAI-1-HT: 2 loci; and tPA-HT: 4 loci. Among these, we identified 4 loci that were associated with both phenotypes individually (at a suggestive *P* value <5 × 10^−3^), thus suggesting that these loci might simultaneously regulate the levels of a hemostatic factor and the risk of suffering from HT after r-tPA.

Table 3 contains the complete information about the 4 loci identified in the multitrait analyses. Briefly, 2 loci in the multitrait analysis between fibrinogen and HT were located in chromosome 3, with lead variants rs34780449 and rs56026866. rs34780449 was located on an intergenic region, 87 kb downstream of the *ROBO1* gene, and rs56026866 is an intronic variant of the *PLXND1* gene. A third locus detected between fibrinogen and HT was located on chromosome 16 with lead variant rs1421067, an intronic variant of the *CHD9* gene. Finally, 1 locus detected in the multitrait analysis between VWF and HT was located on chromosome 5, with lead variant rs10942300, an intronic variant of the *ZNF366* gene.

**FIGURE 2:**
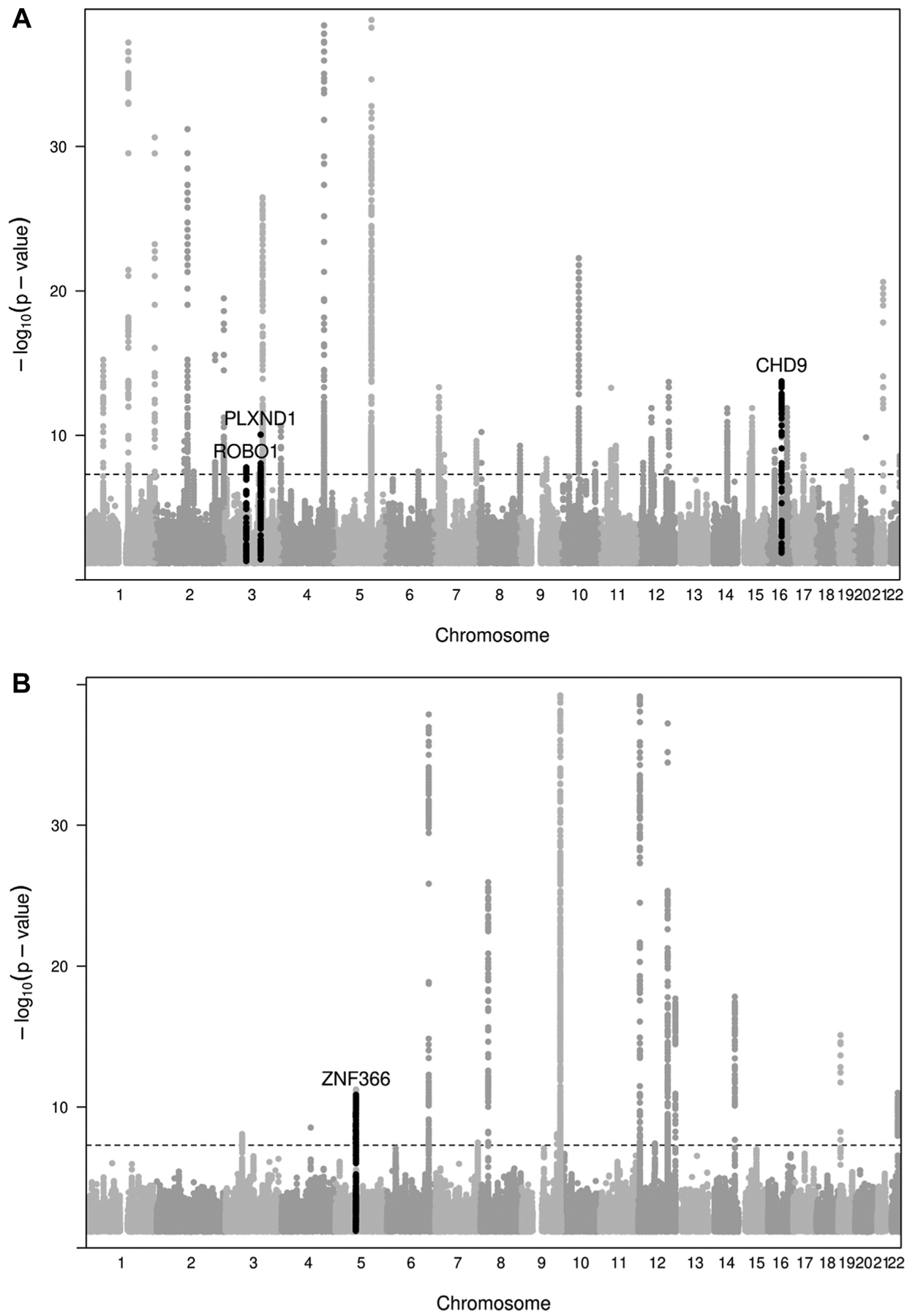
Multitrait analysis. (A) Manhattan plot for fibrinogen–hemorrhagic transformation (HT) multitrait analysis. (B) Manhattan plot for von Willebrand factor–HT multitrait analysis. In orange: newly identified loci associated with HT; in red: newly identified locus associated with fibrinogen and HT.

Manhattan plots with the multiphenotype analysis results between these phenotypes are shown in Figure 2.

### 3.3 | Trait-trait colocalization

**FIGURE 3:**
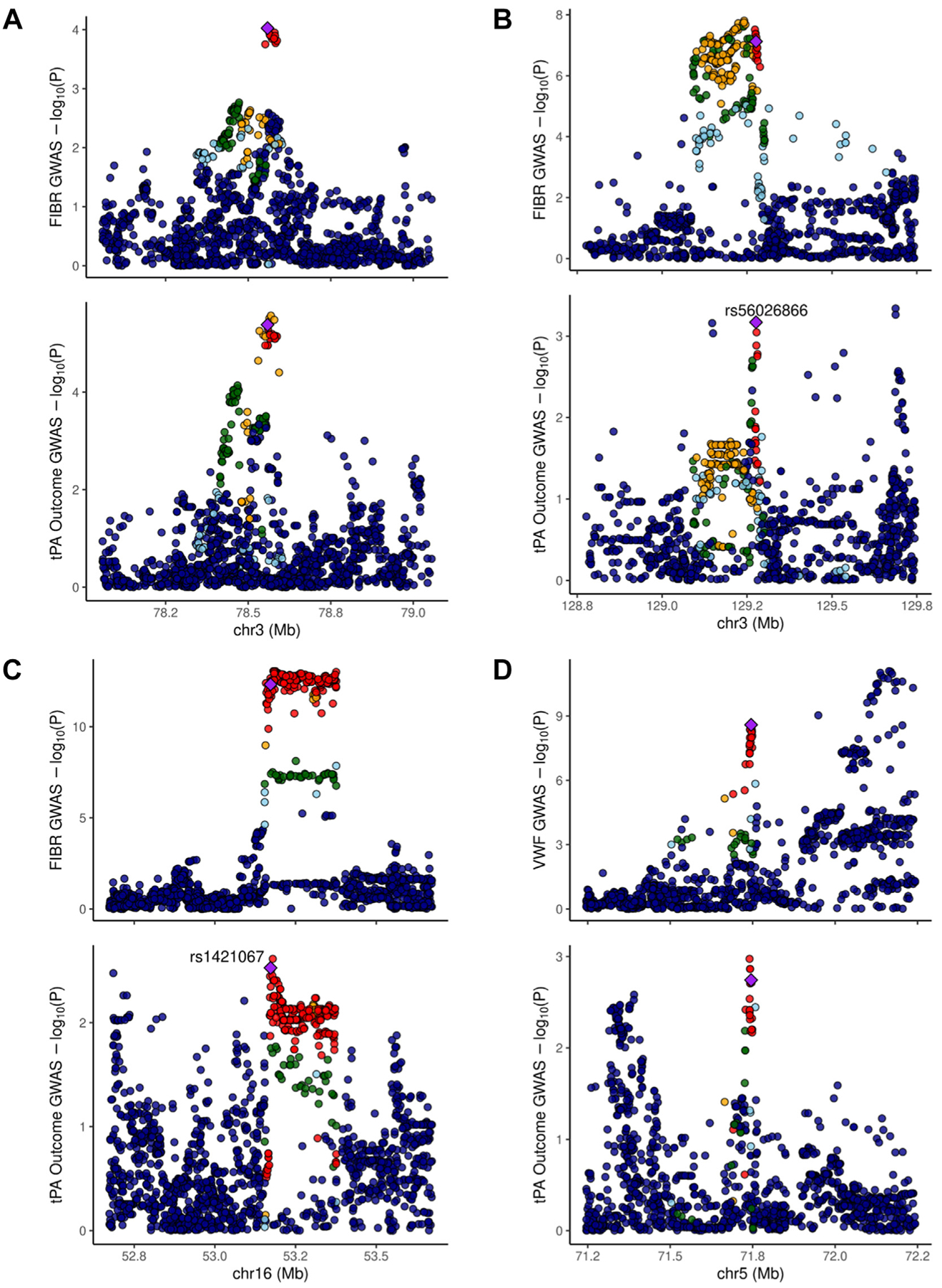
Locus zoom. (A) Colocalization of fibrinogen (FIBR) and recombinant tissue plasminogen activator (tPA) rs34780449 locus. (B) Colocalization of FIBR and recombinant tPA rs56026866 locus. (C) Colocalization of FIBR and recombinant tPA rs1421067 locus. (D) Colocalization of von Willebrand factor and r-tPA rs10942300 locus. Chr, chromosome; GWAS, genome-wide association study.

We performed colocalization in the genomic region with significant local correlation and the 4 significant shared loci. Colocalization results in the region with local correlation between fibrinogen and HT did not suggest the existence of a common regulatory variant (CPC, 0.48). On the other hand, we found significant evidence of colocalization on the chromosome 3 locus identified near *ROBO1* in the multitrait analyses (CPC, 0.96) (Table 3 and Figure 3). This confirms the existence of a common variant that regulates both circulating levels of fibrinogen and HT risk after r-tPA treatment. Colocalization results for the other 3 loci were not significant, although we obtained suggestive results (CPC, >0.6) of the existence of a common regulatory variant for the loci in *PLXND1* and *CHD9* (Table 3).

### 3.4 | MR

In total, 173 genetic instruments from 7 hemostatic factors were used to assess causality with HT risk after r-tPA treatment in patients with IS.

Lower genetically predicted tPA levels were associated with higher risk of HT (OR, 0.57; 95% CI, 0.35–0.93; *P* = .023), but this result was not consistent across all sensitivity methods (Supplementary Table S8 and Supplementary Figure S1). Genetically predicted lower levels of FXI were nominally associated with the risk of HT after r-tPA treatment (OR, 0.87; 95% CI, 0.75–1.00; *P* = .05), with consistent results across sensitivity methods (Supplementary Table S8 and Supplementary Figure S1), but this association was not significant after correction for multiple comparisons. No significant associations were observed with FVII, FVIII, VWF, fibrinogen, or PAI-1, even after removing highly pleiotropic variants in the *ABO* gene (Supplementary Table S8 and Supplementary Figure S1). However, power calculations based on our sample sizes estimated that effect sizes substantially higher than those we are currently observing (OR, 1.08–2.12) are needed for all MR analyses except that of FVII-HT to reach 80% of the power with our sample size. Full statistical power calculations are available in Supplementary Table S4.

## 4 | DISCUSSION

Using large-scale GWAS data from 7 hemostatic traits and HT risk after r-tPA administration in the acute phase of IS, we present indications of potential common regulatory mechanisms between hemostasis and HT. Specifically, we observed a region with shared genetic covariance between plasma levels of fibrinogen and HT after r-tPA treatment. This region located in chromosome 16 contains the *CHD9* gene. A variant in *CHD9* has also been identified in this work jointly associated with HT and fibrinogen, suggesting the existence of common regulatory pathways involved in plasma levels of fibrinogen and HT. *CHD9* codes for the chromodomain helicase DNA-binding protein 9, a DNA-binding protein involved in chromatin regulation and gene transcription [46]. Variants near *CHD9* have been associated with plasma renin activity, a marker for variability in blood pressure (BP) response to antihypertensive agents, and with better systolic BP response to atenolol [47]. BP is an important determinant of functional outcome after r-tPA treatment [48], and its management has been long discussed with regards to r-tPA safety outcomes [49,50].

With regards to the common genes found with the fibrinogen-HT multitrait analyses, the lead variant on chromosome 3 (rs34780449) is an intronic variant located 87 kb downstream *ROBO1*, and the colocalization analysis suggested its regulatory effect on both circulating levels of fibrinogen and HT risk. Gene level association scores for *ROBO1*, calculated using the Multi-marker Analysis of GenoMic Annotation (MAGMA) algorithm [51] and Human Genetic Evidence (HuGE) score [52], available at the Cerebrovascular Disease Knowledge Portal [53], indicate a strong association of common and rare variants in *ROBO1* with hypertension and diastolic and systolic BP.

*ROBO1* encodes for roundabout homolog 1 protein (ROBO1), a receptor of SLIT1 and SLIT2 proteins. Together, they regulate cell migration and are particularly important during neuronal development [52]. The involvement of these proteins with thrombosis regulation is not new since increased *ROBO1* expression has been detected in platelets and megakaryocytes in humans and mice, and SLIT2 acts as a strong regulator of platelet activity and thrombus appearance, prolonging bleeding times [53]. In animal models, ROBO1 has been associated with better recovery after stroke via Slits’ role in angiogenesis and neurogenesis [54]. A reduction in ROBO1 expression levels has also been associated with increased infiltration of polymorphonuclear neutrophils in the brain, which causes an increased inflammatory reaction [55].

*PLXND1* encodes for the Plexin-D1 protein and is mainly linked to regulation of cell migration, development of the nervous system, and regulation of angiogenesis. Plexin-D1 is the receptor of Semaphorin 3E, which together regulate vascular development. A recent study showed that *Plxnd1* knockout mice had worsened neurologic deficits, infarct volume, neuronal survival rate, and blood flow recovery [54]. Semaphorin 3A, a gene in the same family and located in the same chromosome of Semaphorin 3E, was nominally associated (second top hit) with HT in the original GWAS [17], and it has been related to vascular permeability of the blood-brain barrier and brain damage after cerebral ischemia in murine models [55].

Overall, high levels of fibrinogen prior to r-tPA administration have been related to worse clinical response and a 2.7-fold risk of death after r-tPA treatment [56]. On the other hand, it has been observed that fibrinogen depletion after r-tPA administration increases the risk of HT [57,58]. With our sample sizes, we did not have the power to demonstrate a causal effect of genetically determined fibrinogen plasma levels on HT. Although a strong causal relationship between lifelong fibrinogen levels and HT risk has been deemed unlikely, we cannot rule out a causal effect with an effect size below our power threshold. However, overall results indicate that *CHD9*, *ROBO1*, and *PLXND1* would independently regulate fibrinogen levels and HT risk.

Finally, 3 genes were located close to *ZNF366*, *TMEM171*, and *TNPO1* genes in the VWF-HT multitrait analyses. HuGE scores [52] for *ZBF366*, available at the Cerebrovascular Disease Knowledge Portal [53], indicate a moderate association of common and rare variants with systolic BP and pulse pressure. Silencing of *TMEM171* and *TNPO1* genes, also on this locus, has previously proved to result in an increase in VWF levels [29]. *TMEM171* codes for a transmembrane protein that has been associated with different types of cancer [59], and *TNPO1* codes for Transportin, a protein that participates in the nuclear transport of molecules [60]. Neither of these 3 genes has been previously associated with the development or recovery of IS.

We found suggestive evidence for a causal association of higher genetically determined circulating levels of FXI with a decreased risk of HT after tPA treatment. FXI is a serine protease involved in the propagation phase of coagulation and in providing clot stability; a FXI deficiency is related to a mild bleeding disorder [61,62]. Genetically determined FXI levels have been causally associated with increased risk of IS and cardioembolic and undetermined causes of IS [22], but this is the first instance it has been suggested to be related to a higher risk of suffering HT due to r-tPA administration. Our sample size for these phenotypes can allow detection of only effect sizes representing a 30% increase/decrease in risk per every SD increase/decrease in FXI levels. Follow-up studies in larger cohorts are required to validate this observation. FXI has been recently prioritized as a drug target for stroke treatment based on genetic evidence for putative drug effects [62]. If the present results could be confirmed in larger datasets, these data would open an avenue for potential new treatments.

Genetically determined levels of hemostatic factors might not be equivalent to those observed during the stroke acute phase but rather reflect lifetime effects of hemostatic factors on the HT risk given an acute event. However, since the instrument variants are randomly distributed at birth, they are more robust to confounding and reverse causation. Several studies have evaluated the link between prestroke hemostatic factor levels and stroke risk, severity, and outcome as well as levels at admission and after r-tPA treatment [63]. High levels of hemostatic factors before stroke are generally associated with increased risk of stroke. While these might provide a better reflection of the real effect of elevated hemostatic factors on HT in certain situations, the effect of confounding factors or reverse causation on hemostatic levels cannot be ruled out when using protein levels. Unfortunately, our MR study was underpowered to confirm causal associations between lifelong genetically predicted circulating levels of hemostatic factors and HT.

Overall, we detected 4 loci that might regulate both fibrinogen and VWF levels and the risk of HT after r-tPA treatment. Our results suggest the existence of possible common regulatory pathways between levels of fibrinogen and the risk of suffering from HT. Further analyses are warranted to elucidate if these loci affect both phenotypes independently or if there is a mediating effect of plasma levels of fibrinogen on HT risk after r-tPA administration, although our results seem to indicate an independent regulatory effect on both phenotypes. While the direct role of *CHD9*, *ROBO1*, and *ZNF366* in HT is unclear, a mediation effect via its role in BP should be further explored. Finally, we found a suggestive causal effect of genetically determined plasma FXI levels on HT that needs verification in larger samples.

### 4.1 | Strengths and limitations

This is the first study using multiple genetic analysis approaches to interrogate a biological link between genetically predicted plasma levels of hemostatic proteins and the risk of HT due to r-tPA treatment. The small sample size of the r-tPA cohort has limited the results of the MR analysis. However, the appearance of HT after r-tPA treatment is a very specific phenotype, for which very few cohorts are available. The uniqueness of our resource gives added value to these results. Finally, we acknowledge 3 major limitations of this study. First, we only used cohorts of European origin, which could make the results not generalizable to other populations. Second, global correlation estimation methods and the multitrait method used are unable to discriminate between a causal association and an independent association between 2 or more phenotypes. Third, genetically determined levels of hemostatic factors might not reflect hemostatic levels in the acute phase of an IS.

## Supplementary Material

Supplementary Material

Supplementary Table S6

Supplementary Table S7

STROBE_checklist_HT

Supplementary Figure S1

## Figures and Tables

**TABLE 1:** Characteristics and sample sizes of the genome-wide association study datasets used.

Exposure/Outcome	Phenotype	N	Study outcome	Outcome type	Unit	Imputation panel	Citation	URL for data access
Exposure	FVII	20 014	FVII activity levels	Continuous	In(% or IU/mL × 100)	1000 Genomes^a^	PMID: 306429211	https://www.ncbi.nlm.nih.gov/projects/gap/cgi-bin/analysis.cgi?study_id=phs000930.v8.p1&pha=4996
	FVIII	25 897	FVIII activity levels	Continuous	In(% or IU/mL × 100)	1000 Genomes^a^	PMID: 305867372	https://www.ncbi.nlm.nih.gov/projects/gap/cgi-bin/analysis.cgi?study_id=phs000930.v8.p1&pha=4997
	VWF	42 379	VWF levels	Continuous	In(% or IU/mL × 100)	1000 Genomes^a^	PMID: 305867372	https://www.ncbi.nlm.nih.gov/projects/gap/cgi-bin/analysis.cgi?study_id=phs000930.v8.p1&pha=4998
	FXI	16 169	FXI levels	Continuous	In(U/mL)	1000 Genomes^a^	PMID: 280530496	https://ega-archive.org/studies/EGAS00001002123
	Fibrinogen	120 246	Fibrinogen levels	Continuous	In(g/L)	1000 Genomes^a^	PMID: 265615235	https://www.ncbi.nlm.nih.gov/projects/gap/cgi-bin/analysis.cgi?study_id=phs000930.v8.p1&pha=3912
	tPA	26 929	tPA	Continuous	In(ng/mL)	HapMap^b^	PMID: 245783798	https://www.ncbi.nlm.nih.gov/projects/gap/cgi-bin/analysis.cgi?study_id=phs000930.v8.p1&pha=4276
	PAI-1	19 599	PAI-1	Continuous	In(PAI-1 Ag levels [ng/mL] or PAI-1 activity [U/mL])	HapMap^b^	PMID: 229900207	https://www.ncbi.nlm.nih.gov/projects/gap/cgi-bin/analysis.cgi?study_id=phs000930.v8.p1&pha=4275
Outcome	GenoTPA	2045	HT and mortality rates after tPA	Dichotomic	HT-yes (N = 141) vs HT-no (N = 1904)	1000 Genomes^a^	PMID: 33723576	Available upon request

FVII, factor VII; FVIII, factor VIII; FXI, factor XI; GenoTPA, Genetic Study in Ischemic Stroke Patients treated with recombinant tissue plasminogen activator; HT, hemorrhagic transformation; PAI-1, plasminogen activator inhibitor-1; tPA, tissue plasminogen activator; VWF, von Willebrand factor.

a 1000 Genomes Project Consortium [44].

b McCarthy et al. [35].

**TABLE 2:** Local correlations: significant results from local correlation analysis and colocalization.

Phenotype	Chr	Start	End	Rho	Corr	h2_1	h2_2	Var	*P* value	m	PP.H3	PP.H4	CPC
Fibrinogen	16	51703888	53845487	−0.0057	−1.066	0.018244	0.00157	7.3 × 10^−7^	2.45 × 10^−11^	3208	0.27	0.25	0.48
FVII	11	59620206	61870732	0.00871	1.524092	0.00402	0.008126	3.97 × 10^−6^	1.22 × 10^−5^	2796	-	-	-
VWF	12	113030227	113877766	−0.00317	−1.38956	0.011372	0.000459	5.57 × 10^−7^	2.11 × 10^−5^	1128	-	-	-

Chr, chromosome; Corr, correlation value; CPC, conditional probability of colocalization; End, end position of the genomic region; FVII, factor VII; h2_1, estimation of local heritability first trait; h2_2, estimation of local heritability second trait; m, number of single-nucleotide polymorphisms involved in the estimation; PP.H3, posterior probability of hypothesis 3; PP.H4, posterior probability of hypothesis 4; Rho, correlation coefficient; Start, start position of the genomic region; Var, variance of the estimation of local genetic covariance; VWF, von Willebrand factor.

**TABLE 3:** Multitrait and colocalization results.

Phenotype	Chr: position (GRCh37)	rs ID	MAF	Effect 1	Effect2	Effect allele	Nearest gene	*P* value	*P* value, phenotype 1	*P* value, HT after r-tPA	PP.H3	PP.H4	CPC
FIBR-tPA	3:78558745	rs34780449	0.1558	0.0048	0.0608	T	*ROBO*	1.64 × 10^−8^	9.42 × 10^−5^	4.11 × 10^−6^	0.03	0.76	0.96
FIBR-tPA	3:129276412	rs56026866	0.1291	−0.0073	−0.0462	T	*PLXND1*	8.80 × 10^−10^	7.48 × 10^−8^	6.76 × 10^−4^	0.06	0.11	0.67
FIBR-tPA	16:53172643	rsl421067	0.2879	−0.0074	0.0317	T	*CHD9*	1.81 × 10^−14^	4.76 × 10^−13^	2.97 × 10^−3^	0.07	0.13	0.65
VWF-tPA	5:71745412	rsl0942300	0.1289	−0.0027	−0.0426	T	*ZNF366*	2.33 × 10^−10^	2.55 × 10^−9^	1.80 × 10^−3^	0.08	0.01	0.12

Chr, chromosome; CPC, conditional probability of colocalization; Effect1, effect on phenotype 1; Effect2, effect on phenotype 2; FIBR, fibrinogen; HT, hemorrhagic transformation; MAF, minor allele frequency; PP.H3, posterior probability of hypothesis 3; PP.H4, posterior probability of hypothesis 4; r-tPA, recombinant tissue plasminogen activator; tPA, tissue plasminogen activator; VWF, von Willebrand factor.

## APPENDIX

Appendix A contains a list of investigators belonging to the Cohorts for Heart and Aging Research in Genomic Epidemiology Consortium Hemostasis Working Group that contributed to the hemostatic summary data. Abbas Dehghan, Department of Epidemiology and Biostatistics, School of Public Health, Imperial College London, London, UK. Adam S. Heath, Human Genetics Center, Department of Epidemiology, Human Genetics, and Environmental Sciences; School of Public Health, The University of Texas Health Science Center at Houston, Houston, Texas, USA. Alanna C. Morrison, Human Genetics Center, Department of Epidemiology, Human Genetics, and Environmental Sciences; School of Public Health, The University of Texas Health Science Center at Houston, Houston, Texas, USA. Alex P. Reiner, Department of Epidemiology, University of Washington, Seattle, WA, USA. Andrew Johnson, National Heart Lung and Blood Institute, Division of Intramural Research, Population Sciences Branch, The Framingham Heart Study, Framingham, MA, USA. Anne Richmond, MRC Human Genetics Unit, Institute of Genetics and Molecular Medicine, University of Edinburgh, Western General Hospital, Edinburgh, Scotland. Annette Peters, Research Unit Molecular Epidemiology, Helmholtz Zentrum München, München, Germany. Astrid van Hylckama Vlieg, Department of Clinical Epidemiology, Leiden University Medical Center, the Netherlands. Barbara McKnight, Department of Biostatistics, University of Washington, Seattle, WA. Bruce M. Psaty, Cardiovascular Health Research Unit, Department of Medicine, University of Washington, Seattle, WA, United States. Caroline Hayward, MRC Human Genetics Unit, Institute of Genetics and Molecular Medicine, University of Edinburgh, Western General Hospital, Edinburgh, Scotland. Cavin Ward-Caviness, Office of Research and Development, U.S. Environmental Protection Agency, Chapel Hill, NC, USA. Christopher O’Donnell, Cardiology, VA Boston Healthcare System, Boston, MA, USA. Daniel Chasman, Division of Preventive Medicine, Brigham and Women’s Hospital, Boston, MA, USA. David P. Strachan, Population Health Research Institute, St George’s University of London, London, UK. David A. Tregouet, Bordeaux Population Health Research Center, University of Bordeaux, Bordeaux, France. Dennis Mook-Kanamori, Department of Clinical Epidemiology, Leiden University Medical Center, Leiden, the Netherlands. Dipender Gill, Department of Epidemiology and Biostatistics, School of Public Health, Imperial College London, London, UK. Florian Thibord, National Heart Lung and Blood Institute, Division of Intramural Research, Population Sciences Branch, The Framingham Heart Study, Framingham, MA, USA. Folkert W. Asselbergs, Department of Cardiology, Division Heart & Lungs, University Medical Center Utrecht, Utrecht University, Utrecht, the Netherlands. Frank W.G. Leebeek, Department of Hematology, Erasmus Medical Center, Rotterdam, the Netherlands. Frits R. Rosendaal, Department of Clinical Epidemiology, Leiden University Medical Center, Leiden, the Netherlands. Gail Davies, Lothian Birth Cohorts, Department of Psychology, University of Edinburgh, Edinburgh, UK. Georg Homuth, Department of Functional Genomics, Interfaculty Institute for Genetics and Functional Genomics, University Medicine Greifswald, Greifswald, Germany. Gerard Temprano, Unit of Genomics of Complex Diseases. Institut de Recerca Sant Pau (IR-Sant Pau), Barcelona, Spain. Harry Campbell, Global Health Research, Usher Institute for Population Health Sciences and Informatics, University of Edinburgh, Edinburgh, Scotland, UK. Herman A. Taylor, The Institute for Translational Genomics and Population Sciences, Department of Pediatrics, The Lundquist Institute for Biomedical Innovation at Harbor-UCLA Medical Center, Torrance, CA USA. Jan Bressler, Human Genetics Center, Department of Epidemiology, Human Genetics, and Environmental Sciences; School of Public Health, The University of Texas Health Science Center at Houston, Houston, Texas, USA. Jennifer E. Huffman, Massachusetts Veterans Epidemiology Research and Information Center (MAVERIC), VA Boston Healthcare System, Boston, MA, USA. Jerome I. Rotter, The Institute for Translational Genomics and Population Sciences, Department of Pediatrics, The Lundquist Institute for Biomedical Innovation at Harbor-UCLA Medical Center, Torrance, CA USA. Jie Yao, The Institute for Translational Genomics and Population Sciences, Department of Pediatrics, The Lundquist Institute for Biomedical Innovation at Harbor-UCLA Medical Center, Torrance, CA USA. James F. Wilson, MRC Human Genetics Unit, Institute of Genetics and Cancer, University of Edinburgh, Western General Hospital, Edinburgh, UK. Joshua C. Bis, Cardiovascular Health Research Unit Department of Medicine University of Washington Seattle Washington USA. Julie M. Hahn, Human Genetics Center, Department of Epidemiology, Human Genetics, and Environmental Sciences; School of Public Health, The University of Texas Health Science Center at Houston, Houston, Texas, USA. Karl C. Desch, Department of Pediatrics, University of Michigan, C.S. Mott Children’s Hospital, Ann Arbor, MI, USA. Kerri L. Wiggins, Cardiovascular Health Research Unit Department of Medicine University of Washington Seattle Washington USA. Laia Díez-Ahijado, Unit of Genomics of Complex Diseases. Institut de Recerca Sant Pau (IR-Sant Pau), Barcelona, Spain. Laura M. Raffield, Department of Genetics, University of North Carolina at Chapel Hill, Chapel Hill, NC, USA. Lawrence F. Bielak, Department of Epidemiology, School of Public Health, University of Michigan, Ann Arbor, MI. Lisa R. Yanek, Department of Medicine, Johns Hopkins University School of Medicine, Baltimore, MD, USA. Marcus E. Kleber, SYNLAB MVZ für Humangenetik Mannheim, Mannheim, Germany. Maria Sabater-Lleal, Unit of Genomics of Complex Diseases. Institut de Recerca Sant Pau (IR-Sant Pau), Barcelona, Spain. Martina Mueller, Research Unit Molecular Epidemiology, Helmholtz Zentrum München, München, Germany. Maryam Kavousi, Department of Epidemiology, Erasmus Medical Center, University Medical Center Rotterdam, Rotterdam, Netherlands. Massimo Mangino, Department of Twin Research and Genetic Epidemiology, Kings College London, London, UK. Matthew P. Conomos, Department of Biostatistics, University of Washington, Seattle, WA, USA. Melissa Liu, National Heart Lung and Blood Institute, Division of Intramural Research, Population Sciences Branch, The Framingham Heart Study, Framingham, MA, USA. Michael R. Brown, Human Genetics Center, Department of Epidemiology, Human Genetics, and Environmental Sciences; School of Public Health, The University of Texas Health Science Center at Houston, Houston, Texas, USA. Min-A Jhun, Department of Epidemiology, University of Michigan School of Public Health, Ann Arbor, MI, USA. Ming-Huei Chen, Population Sciences Branch, National Heart, Lung, and Blood Institute, Framingham, Massachusetts, USA. Moniek P.M. de Maat, Department of Hematology, Erasmus MC University Medical Center, The Netherlands. Nathan Pankratz, Department of Laboratory Medicine and Pathology, University of Minnesota Medical School, Minneapolis, MN, USA. Nicholas L. Smith, Department of Epidemiology, University of Washington, Seattle, WA, USA. Patricia A. Peyser, Department of Epidemiology, School of Public Health, University of Michigan, Ann Arbor, MI, USA. Paul Elliot, Department of Epidemiology and Biostatistics, School of Public Health, Imperial College London, London, UK. Paul S. de Vries, Human Genetics Center, Department of Epidemiology, Human Genetics, and Environmental Sciences; School of Public Health, The University of Texas Health Science Center at Houston, Houston, Texas, USA. Peng Wei, Department of Biostatistics, The University of Texas MD Anderson Cancer Center, Houston. Philipp S. Wild, Department of Cardiology, Cardiology I, University Medical Center, Johannes Gutenberg University Mainz, Mainz, Germany. Pierre E. Morange, Hematology Laboratory, La Timone University Hospital of Marseille, Marseille, France. Pim van der Harst, Department of Cardiology, University Medical Center Utrecht, Utrecht, Netherlands. Qiong Yang, Department of Biostatistics, Boston University School of Public Health, Boston, MA, USA. Riccardo Marioni, Centre for Genomic and Experimental Medicine, Institute of Genetics and Molecular Medicine, University of Edinburgh, Edinburgh, UK. Ruifang Li, Department of Clinical Epidemiology, Leiden University Medical Center, the Netherlands. Scott M. Damrauer, Department of Surgery, Perelman School of Medicine, University of Pennsylvania, Philadelphia, PA, USA. Simon R. Cox, Department of Psychology, University of Edinburgh, Edinburgh, UK. Stella Trompet, Section of Gerontology and Geriatrics, Department of Internal Medicine, Leiden University Medical Center, Leiden, The Netherlands. Stephan B. Felix, Department of Internal Medicine B, University Medicine Greifswald, Greifswald, Germany. Uwe Völker, Department of Functional Genomics, University Medicine Greifswald, Greifswald, Germany. Weihong Tang, School of Public Health, University of Minnesota, Minneapolis, MN, USA. Wolfgang Koenig, DZHK (German Centre for Cardiovascular Research), partner site Munich Heart Alliance, Munich, Germany. J. Wouter Jukema, Department of Cardiology, Leiden University Medical Center, The Netherlands. Xiuqing Guo, The Institute for Translational Genomics and Population Sciences, Department of Pediatrics, The Lundquist Institute for Biomedical Innovation at Harbor-UCLA Medical Center, Torrance, CA USA.
