# Supplementary material for: A multitrait genetic study of hemostatic factors and hemorrhagic transformation after stroke treatment
Source: J Thromb Haemost. Author manuscript; Available in PMC 2024 May 20. (PMC11103592; doi:10.1016/j.jtha.2023.11.027)
Supplement: Supplementary Material [file NIHMS1993459-supplement-Supplementary_Material.docx]

**A multi-trait genetic Study of Hemostatic factors and Hemorrhagic transformation after stroke treatment.**

Cristina Gallego-Fabrega^1^; Gerard Temprano-Sagrera^2^; Jara Cárcel-Márquez^1^; Elena Muiño^1^; Natalia Cullell^1,3^; Miquel Lledós^1^; Laia Llucià-Carol^1^; Jesús M. Martin-Campos^1^, Tomás Sobrino^4^, Emilio Rodríguez-Castro^5^, Mònica Millán^6^, Lucía Muñoz-Narbona^6^, Elena López-Cancio^7^, Marc Ribó^8^, José Álvarez-Sabín^90^, Jordi Jiménez-Conde^10^, Jaume Roquer^10^, Silvia Tur^11^, Victor Obach^12^, Juan Francisco-Arenillas^13^, Tomás Segura^14^, Gemma Serrano-Heras^15^, Joan Martí-Fàbregas^16^, M. Mar Freijo-Guerrero^17^, Francisco Moniche^18^, Mar Castellanos^19^, Alanna C. Morrison^20^, Nicholas L Smith^21,22,23^, Paul S de Vries^20^, Israel Fernández-Cadenas^1^*, Maria Sabater-Lleal^2,24^*. Cohorts for Heart and Aging Research in Genomic Epidemiology Consortium (CHARGE) and the Spanish Stroke Genetic Consortium.

1 Stroke Pharmacogenomics and Genetics Group, Institut de Recerca Sant Pau (IR SANT PAU), Barcelona, Spain.

2 Genomics of Complex Disease Group, Institut de Recerca Sant Pau (IR SANT PAU), Barcelona, Spain.

3 Neurology Unit, Hospital Universitari MútuaTerrassa. Terrassa, Spain.

4 Clinical Neurosciences Research Laboratories, Health Research Institute of Santiago de

Compostela (IDIS), Santiago de Compostela, Spain.

5 Department of Neurology, Hospital Clínico Universitario de Santiago (CHUS), Santiago de Compostela, Spain.

6 Department of Neuroscience, Hospital Universitario Hermanos Trias y Pujol (HUGTP), Badalona, Spain.

7 Stroke Unit, Neurology Department, Hospital Universitario Central de Asturias (HUCA), Instituto de Investigación Sanitaria del Principado de Asturias (ISPA), Oviedo, Spain.

8 Stroke Unit, Hospital Universitario Valle de Hebrón (HUVH), Barcelona, Spain.

9 Department of Neurology, Hospital Universitario Valle de Hebrón (HUVH), Universitat Autònoma de Barcelona (UAB), Barcelona, Spain.

10 Department of Neurology, Neurovascular Research Group, Instituto de investigaciones médicas Hospital del Mar (IMIM) Hospital del Mar, Barcelona, Spain.

11 Department of Neurology, Hospital Universitario Son Espases (HUSE), Mallorca, Spain.

12 Department of Neurology, Hospital Clínic i Provincial de Barcelona, Barcelona, Spain.

13 Department of Neurology, Hospital Clínico Universitario, University of Valladolid, Valladolid, Spain.

14 Department of Neurology, Complejo Hospitalario Universitario de Albacete (CHUA), Universidad de Castilla-La Mancha (UCLM), Albacete, Spain15 Research Unit, Complejo Hospital Universitario de Albacete (CHUA), Albacete, Spain.

16 Department of Neurology, Hospital de la Santa Creu i Sant Pau, IIB-Sant Pau, Barcelona, Spain.

17 Neurovascular Unit, Biocruces Bizkaia Health Research Institute, Bilbao, Spain.

18 Department of Neurology, Hospital Universitario Virgen del Rocio, Instituto de Biomedicina de Sevilla (IBIS), Seville, Spain.

19 Department of Neurology, Hospital Universitario de A Coruña (CHUAC), Biomedical Research Institute, A Coruña, Spain.

20 Human Genetics Center, Department of Epidemiology, Human Genetics, and Environmental Sciences, School of Public Health, The University of Texas Health Science Center at Houston, Houston, USA.

21 Department of Epidemiology, University of Washington, Seattle, WA, USA.

22 Kaiser Permanente Washington Health Research Institute, Kaiser Permanente Washington, Seattle, WA, USA.

23 Department of Veterans Affairs Office of Research and Development, Seattle Epidemiologic Research and Information Center, Seattle, WA, USA.

24 Cardiovascular Medicine Unit, Department of Medicine, Karolinska Institutet, Stockholm, Sweden

*These authors contributed equally as last authors

Short title: Genetics of 7 Hemostatic factors and HT

Corresponding Authors:

Israel Fernández-Cadenas, PhD

Stroke Pharmacogenomics and Genetics.

Institut de Recerca Sant Pau (IR SANT PAU), Sant Quintí 77-79, 08041 Barcelona, Spain

Email [israelcadenas@yahoo.es](mailto:israelcadenas@yahoo.es)

Maria Sabater Lleal, PhD

Genomics of Complex Disease Group.

Institut de Recerca Sant Pau (IR SANT PAU), Sant Quintí 77-79, 08041 Barcelona, Spain

Email [msabater@santpau.cat](mailto:msabater@santpau.cat)

**SUPPLEMENTARY METHODS:**

**GWAS data sources**

*The GenoTPA cohort consisted of AIS patients admitted to an emergency department and treated with intravenous rtPA, within 4.5 hours of symptom onset. Exclusion criteria included patients under 18 years old, patients with remote parenchymal hematoma, missing hemorrhagic transformation (HT) information, onset to treatment time over 4.5 hours, and patients who had undergone endovascular therapy. HT was assessed in a follow-up CT scan and radiologically classified according to the European Cooperative Acute Stroke Study (ECASS) criteria. Demographic data, medical history, cardiovascular risk factors, clinical examination, stroke severity assessed with the National Institutes of Health Stroke Score (NIHSS) at initial evaluation, and treatment decisions were obtained from the medical records. The HT vs. non-HT association analysis was performed in SNPTEST v2.5.216, under an additive genetic model adjusted for age, sex, three principal components (PCs) and clinical variables associated with PH after a logistic regression: baseline NIHSS and Diabetes (p <0.05). Extended information on this study can be found in Carrera et al. Brain. 2021 original paper.*

**Genetic correlation and heritability estimates**

Pre-computed LD scores from the European population of 1000 Genomes project phase III^1^ were used. To ensure that all variants were well-imputed in most studies, the datasets were restricted to a list of 1,217,311 SNPs^2^ from HapMap3^3^.

For the local correlations calculated with SUPERGNOVA, we used the genome partitions obtained with LDetect^4^ from the 1000 Genomes Project data^5^, from European ancestry, to define the regions.

**Trait-trait colocalization**

For these analyses, loci were defined as +/- 500 kb around the lead variant. To consider a locus as pleiotropic, we used posterior probability of hypothesis (PPH) values in the formula PPH4/(PPH3+PPH4), to create a conditional probability of colocalization (CPC) that gives the probability that a common regulatory variant exists, assuming the existence of a signal in both traits, where PPH3 indicates the probability that the locus is associated with both traits without evidence of the existence of a common regulator variant, and PPH4 returns the probability of this association occurring due to a common genetic variant, considering LD patterns.

**Mendelian Randomization**

Sensitivity MR approaches were applied: MR-Egger^6^, weighted median and penalized weighted median. Horizontal pleiotropy was assessed using Egger regression^6^ and heterogeneity was analyzed with Cochran Q statistic^7^. Finally, when significant MR results were found, we used the MR-PRESSO (Mendelian Randomization Pleiotropy Residual Sum and Outlier) outlier test^8^ and the leave-one-out analysis to explore for the presence of outliers that could bias the results.

MR power calculation:

We used the method described by Deng *et al.*^9^ to calculate the minimum detectable odds ratio (OR) with an 80 % of power, considering our conditions of sample size and proportion of variance explained by the instruments (Supplementary Table S4).

**Bibliography**

1. 1000 Genomes Project Consortium, Auton A, Brooks LD, Durbin RM, Garrison EP, Kang HM, et al. A global reference for human genetic variation. *Nature* [Internet]. 2015;526:68–74. Available from: http://www.nature.com/articles/nature15393

2. Bulik-Sullivan B, Loh PR, Finucane HK, Ripke S, Yang J, Patterson N, et al. LD score regression distinguishes confounding from polygenicity in genome-wide association studies. *Nat. Genet.* [Internet]. 2015 [cited 2022 Mar 1];47:291–295. Available from: /pmc/articles/PMC4495769/

3. Altshuler DM, Gibbs RA, Peltonen L, Schaffner SF, Yu F, Dermitzakis E, et al. Integrating common and rare genetic variation in diverse human populations. *Nature* [Internet]. 2010 [cited 2022 Mar 11];467:52–58. Available from: /labs/pmc/articles/PMC3173859/

4. Berisa T, Pickrell JK. Approximately independent linkage disequilibrium blocks in human populations. *Bioinformatics* [Internet]. 2016 [cited 2022 Sep 30];32:283. Available from: /pmc/articles/PMC4731402/

5. Altshuler DM, Durbin RM, Abecasis GR, Bentley DR, Chakravarti A, Clark AG, et al. An integrated map of genetic variation from 1,092 human genomes. *Nat. 2012 4917422* [Internet]. 2012 [cited 2022 Sep 30];491:56–65. Available from: https://www.nature.com/articles/nature11632

6. Rees JMB, Wood AM, Burgess S. Extending the MR-Egger method for multivariable Mendelian randomization to correct for both measured and unmeasured pleiotropy. *Stat. Med.* [Internet]. 2017 [cited 2022 Jun 15];36:4705–4718. Available from: https://pubmed.ncbi.nlm.nih.gov/28960498/

7. Higgins JPT, Thompson SG. Quantifying heterogeneity in a meta-analysis. *Stat. Med.* [Internet]. 2002 [cited 2022 Jun 15];21:1539–1558. Available from: https://pubmed.ncbi.nlm.nih.gov/12111919/

8. Verbanck M, Chen CY, Neale B, Do R. Detection of widespread horizontal pleiotropy in causal relationships inferred from Mendelian randomization between complex traits and diseases. *Nat. Genet. 2018 505* [Internet]. 2018 [cited 2022 Feb 21];50:693–698. Available from: https://www.nature.com/articles/s41588-018-0099-7

9. Deng L, Zhang H, Yu K. Power calculation for the general two-sample Mendelian randomization analysis. *Genet. Epidemiol.* [Internet]. 2020 [cited 2022 Sep 30];44:290. Available from: /pmc/articles/PMC8766247/

**SUPPLEMENTARY TABLES:**

**Supplementary Table S1**: GenoTPA cohort characteristics.

|  |  | **PH** | |  |  |
| --- | --- | --- | --- | --- | --- |
|  | **TOTAL (n=1,324)** | **Absence (n=1,253)** | **Presence (n=71)** | **p-value** | **OR  (95%CI)** |
| Sex, male (%) | 733 (55.4) | 692 (55.2) | 41 (57,7) | 0.7 | 1.11 (0.68–1.79) |
| AF (%) | 361 (27.3) | 336 (26.9) | 25 (35.2) | 0.13 | 1.11 (0.68–1.79) |
| DM (%) | 329 (24.8) | 302 (24.2) | 26 (36.6) | 0.02* | 1.11 (0.68–1.79) |
| HTN (%) | 867 (65.8) | 817 (65.5) | 50 (71.4) | 0.31 | 1.11 (0.68–1.79) |
| ST (%) | 374 (34.5) | 356 (34.7) | 18 (31) | 0.57 | 1.11 (0.68–1.79) |
| **TOAST (%)** |  |  |  |  |  |
| *CE* | 556 (43.4) | 518 (42.6) | 26 (36.6) | 0.04* | 1.65 (1.02–2.69) |
| *LAA* | 219 (17.1) | 215 (17.7) | 4 (5.6) | 0.01* | 0.29 (0.07–0.79) |
| *SVO* | 56 (4.4) | 55 (4.5) | 1 (1.5) | 0.36 | 0.31 (0.01–1.89) |
| *OT* | 24 (1.9) | 23 (1.9) | 1 (1.5) | 0.1 | 0.77 (0.02–4.91) |
| *UND* | 426 (33.3) | 402 (33.1) | 24 (35.3) | 0.71 | 1.1 (0.6–1.8) |
| Age, years (IQR) | 70 (70-82) | 75 (45-82) | 77 (70-82) | 0.28 |  |
| Baseline NIHSS (IQR) | 12 (7-18) | 11 (7-18) | 18 (14-22) | <0.0001* |  |
| Glucose, mg/dl (IQR) | 120 (103-147) | 119 (103-146) | 130 (105-168) | 0.18 |  |
| OTT, min (IQR) | 130 (90-180) | 128 (90-180) | 140 (90-180) | 0.54 |  |
| SPB, mmHg (IQR) | 155 (138-172) | 154 (138-172) | 158 (144-173) | 0.36 |  |
| DBP, mm HG (IQR) | 82 (71-98) | 81 (71-97) | 85 (70-112) | 0.34 |  |

**Supplementary Table S2**: Genetic variants selected for MR Analysis.

| **Phenotype** | **Number of Tests** | **Statistical Threshold** |
| --- | --- | --- |
| FVII | 2330 | 2.20E-05 |
| FVIII | 2330 | 2.20E-05 |
| VWF | 2330 | 2.20E-05 |
| Fibrinogen | 2328 | 2.20E-05 |
| FXI | 2330 | 2.20E-05 |
| PAI | 2294 | 2.20E-05 |
| TPA | 2296 | 2.20E-05 |

**Supplementary Table S3**: Genetic variants selected for MR Analysis.

| **Exposure** | **Variants  p-value<5x10-8** | **Variants   after clumping** | **Variants in  HT GWAS** |
| --- | --- | --- | --- |
| FVII | 1782 | 32 | 16 |
| FVIII | 1380 | 42 | 28 |
| FXI | 1390 | 25 | 15 |
| VWF | 2631 | 72 | 46 |
| Fibrinogen | 5076 | 73 | 61 |
| PAI1 | 24 | 4 | 4 |
| tPA | 96 | 3 | 3 |

**Supplementary Table S4**: MR Power Calculations.

|  | **Detectable Odds Ratio** | | | | | | |
| --- | --- | --- | --- | --- | --- | --- | --- |
| **Phenotype** | **Fibrinogen (R2 = 2.6%)** | **FVII**  **(R2 = 17.6%)** | **FVIII**  **(R2 = 17%)** | **VWF**  **(R2 = 21.3%)** | **tPA**  **(R2 = 0.75%)** | **PAI-1**  **(R2 = 1.26%)** | **FXI**  **(R2 = 5.89%)** |
| **GenotPA (n = 1904)** | 1.49 | 1.08 | 1.17 | 1.15 | 2.12 | 1.36 | 1.3 |
| R2: Variance explained by selected variants | | | | | | | |

**Supplementary Table S5**: Global Genetic Correlations results.

|  |  | **Genetic Correlation with HT** | | |
| --- | --- | --- | --- | --- |
| **Phenotype** | **h2** | **Correlation** | **p-value** | **q-value** |
| **FVII** | 0.19 | 0.02 | 0.92 | 0.97 |
| **FVIII** | 0.10 | 0.08 | 0.56 | 0.82 |
| **VWF** | 0.14 | 0.13 | 0.24 | 0.82 |
| **FXI** | 0.17 | 0.43 | 0.29 | 0.82 |
| **Fibrinogen** | 0.12 | -0.04 | 0.58 | 0.82 |
| **PAI-1** | 0.07 | -0.01 | 0.97 | 0.97 |
| **tPA** | 0.07 | 0.14 | 0.41 | 0.82 |

**Supplementary Table S6**: Local Genetic Correlations results.

*Attaches as a separated .pdf file, available as a .xlsx file*

**Supplementary Table S7**: Significant results of Multi-trait analysis. (*) denotes novel loci for one or both of the traits analyzed.

*Attaches as a separated .pdf file, available as a .xlsx file*

**Supplementary Table S8**: MR Analysis results. (*) denotes nominal results.

| **Exposure** | **Outcome** | **MR Method** | **n SNPs** | **b** | **se (95% CI)** | **or (95% CI)** | **p-value** | **q-value** | |  |
| --- | --- | --- | --- | --- | --- | --- | --- | --- | --- | --- |
| FVII | PH vs. no-TH | Inverse variance weighted | 16 | -0.16 | 0.16 (-0.48 - 0.16) | 0.85 (0.62 - 1.17) | 0.32 | 1.00 |  |  |
|  |  | MR Egger | 16 | -0.05 | 0.30 (-0.65 - 0.54) | 0.95 (0.52 - 1.72) | 0.86 | 1.00 |  |  |
|  |  | Weighted median | 16 | -0.1 | 0.15 (-0.39 - 0.19) | 0.90 (0.68 - 1.21) | 0.50 | 1.00 |  |  |
|  |  | Penalised weighted median | 16 | -0.07 | 0.15 (-0.35 - 0.22) | 0.94 (0.70 - 1.25) | 0.65 | 1.00 |  |  |
| FVIII | PH vs. no-TH | Inverse variance weighted | 28 | 0.07 | 0.05 (-0.03 - 0.17) | 1.07 (0.97 - 1.19) | 0.18 | 0.68 |  |  |
|  |  | MR Egger | 28 | 0.11 | 0.09 (-0.06 - 0.28) | 1.12 (0.94 - 1.33) | 0.23 | 0.68 |  |  |
|  |  | Weighted median | 28 | 0.06 | 0.07 (-0.08 - 0.20) | 1.06 (0.92 - 1.22) | 0.42 | 0.85 |  |  |
|  |  | Penalised weighted median | 28 | 0.02 | 0.07 (-0.12 - 0.16) | 1.02 (0.89 - 1.17) | 0.78 | 1.00 |  |  |
| FXI | PH vs. no-TH | **Inverse variance weighted** | **15** | **-0.14** | **0.07 (-0.29 - 0.00)** | **0.87 (0.75 - 1.00)** | **0.05** | **0.11** | * |  |
|  |  | MR Egger | 15 | -0.09 | 0.14 (-0.36 - 0.19) | 0.92 (0.70 - 1.20) | 0.55 | 0.82 |  |  |
|  |  | Weighted median | 15 | -0.22 | 0.10 (-0.42 - -0.02) | 0.80 (0.66 - 0.98) | 0.03 | 0.09 |  |  |
|  |  | Penalised weighted median | 15 | -0.22 | 0.10 (-0.41 - -0.02) | 0.80 (0.66 - 0.98) | 0.03 | 0.09 |  |  |
| VWF | PH vs. no-TH | Inverse variance weighted | 46 | 0.03 | 0.05 (-0.06 - 0.12) | 1.03 (0.94 - 1.13) | 0.55 | 0.83 |  |  |
|  |  | MR Egger | 46 | 0.1 | 0.07 (-0.05 - 0.24) | 1.10 (0.96 - 1.28) | 0.18 | 0.50 |  |  |
|  |  | Weighted median | 46 | 0.07 | 0.06 (-0.05 - 0.19) | 1.07 (0.95 - 1.21) | 0.25 | 0.50 |  |  |
|  |  | Penalised weighted median | 46 | 0.09 | 0.06 (-0.03 - 0.21) | 1.09 (0.97 - 1.23) | 0.14 | 0.50 |  |  |
| Fibrinogen | PH vs. no-TH | Inverse variance weighted | 61 | 0.04 | 0.19 (-0.33 - 0.41) | 1.04 (0.72 - 1.50) | 0.84 | 1.00 |  |  |
|  |  | MR Egger | 61 | 0 | 0.41 (-0.79 - 0.80) | 1.00 (0.45 - 2.23) | 0.99 | 1.00 |  |  |
|  |  | Weighted median | 61 | 0.19 | 0.29 (-0.38 - 0.76) | 1.21 (0.68 - 2.14) | 0.52 | 1.00 |  |  |
|  |  | Penalised weighted median | 61 | 0.19 | 0.29 (-0.38 - 0.76) | 1.21 (0.68 - 2.15) | 0.52 | 1.00 |  |  |
| PAI1 | PH vs. no-TH | Inverse variance weighted | 4 | 0.02 | 0.08 (-0.15 - 0.18) | 1.02 (0.86 - 1.20) | 0.85 | 1.00 |  |  |
|  |  | MR Egger | 4 | 0.27 | 0.42 (-0.55 - 1.09) | 1.31 (0.58 - 2.99) | 0.58 | 1.00 |  |  |
|  |  | Weighted median | 4 | -0.07 | 0.10 (-0.26 - 0.13) | 0.94 (0.77 - 1.14) | 0.51 | 1.00 |  |  |
|  |  | Penalised weighted median | 4 | -0.07 | 0.10 (-0.27 - 0.14) | 0.94 (0.76 - 1.15) | 0.52 | 1.00 |  |  |
| tPA | PH vs. no-TH | **Inverse variance weighted** | **3** | **-0.56** | **0.25 (-1.05 - -0.07)** | **0.57 (0.35 - 0.93)** | **0.02** | **0.06** | * |  |
|  |  | MR Egger | 3 | 0.29 | 1.24 (-2.14 - 2.72) | 1.34 (0.12 - 15.21) | 0.85 | 1.00 |  |  |
|  |  | Weighted median | 3 | -0.63 | 0.28 (-1.17 - -0.09) | 0.53 (0.31 - 0.91) | 0.02 | 0.06 |  |  |
|  |  | Penalised weighted median | 3 | -0.63 | 0.29 (-1.21 - -0.05) | 0.53 (0.30 - 0.95) | 0.03 | 0.06 |  |  |
| After removing *ABO* variants | | | |  |  |  |  |  |  |  |
| FVIII | PH vs. no-TH | Inverse variance weighted | 25 | 0.04 | 0.06 (-0.08 - 0.17) | 1.04 (0.92 - 1.18) | 0.51 | 0.76 |  |  |
|  |  | MR Egger | 25 | 0.08 | 0.10 (-0.12 - 0.28) | 1.08 (0.89 - 1.33) | 0.44 | 0.76 |  |  |
|  |  | Weighted median | 25 | 0.08 | 0.09 (-0.10 - 0.26) | 1.08 (0.91 - 1.29) | 0.38 | 0.76 |  |  |
|  |  | Penalised weighted median | 25 | 0.08 | 0.08 (-0.09 - 0.24) | 1.08 (0.92 - 1.28) | 0.35 | 0.76 |  |  |
| VWF | PH vs. no-TH | Inverse variance weighted | 44 | -0.02 | 0.05 (-0.12 - 0.08) | 0.98 (0.89 - 1.08) | 0.67 | 1.00 |  |  |
|  |  | MR Egger | 44 | 0.03 | 0.08 (-0.13 - 0.19) | 1.03 (0.88 - 1.20) | 0.72 | 1.00 |  |  |
|  |  | Weighted median | 44 | 0.04 | 0.06 (-0.09 - 0.16) | 1.04 (0.92 - 1.18) | 0.55 | 1.00 |  |  |
|  |  | Penalised weighted median | 44 | 0.04 | 0.06 (-0.08 - 0.17) | 1.05 (0.92 - 1.19) | 0.49 | 1.00 |  |  |
| * Nominal associations | |  |  |  |  |  |  |  | |  |

**Supplementary Figure S1**: MR Analysis results. (A) Forest Plot representing the results from MR analyses. (B) Scatter Plot FXI-HT MR analysis. (C) Scatter Plot tPA-HT MR analysis.
