# Supplementary figures and images for: A multitrait genetic study of hemostatic factors and hemorrhagic transformation after stroke treatment
Source: J Thromb Haemost. Author manuscript; Available in PMC 2024 May 20. (PMC11103592; doi:10.1016/j.jtha.2023.11.027)

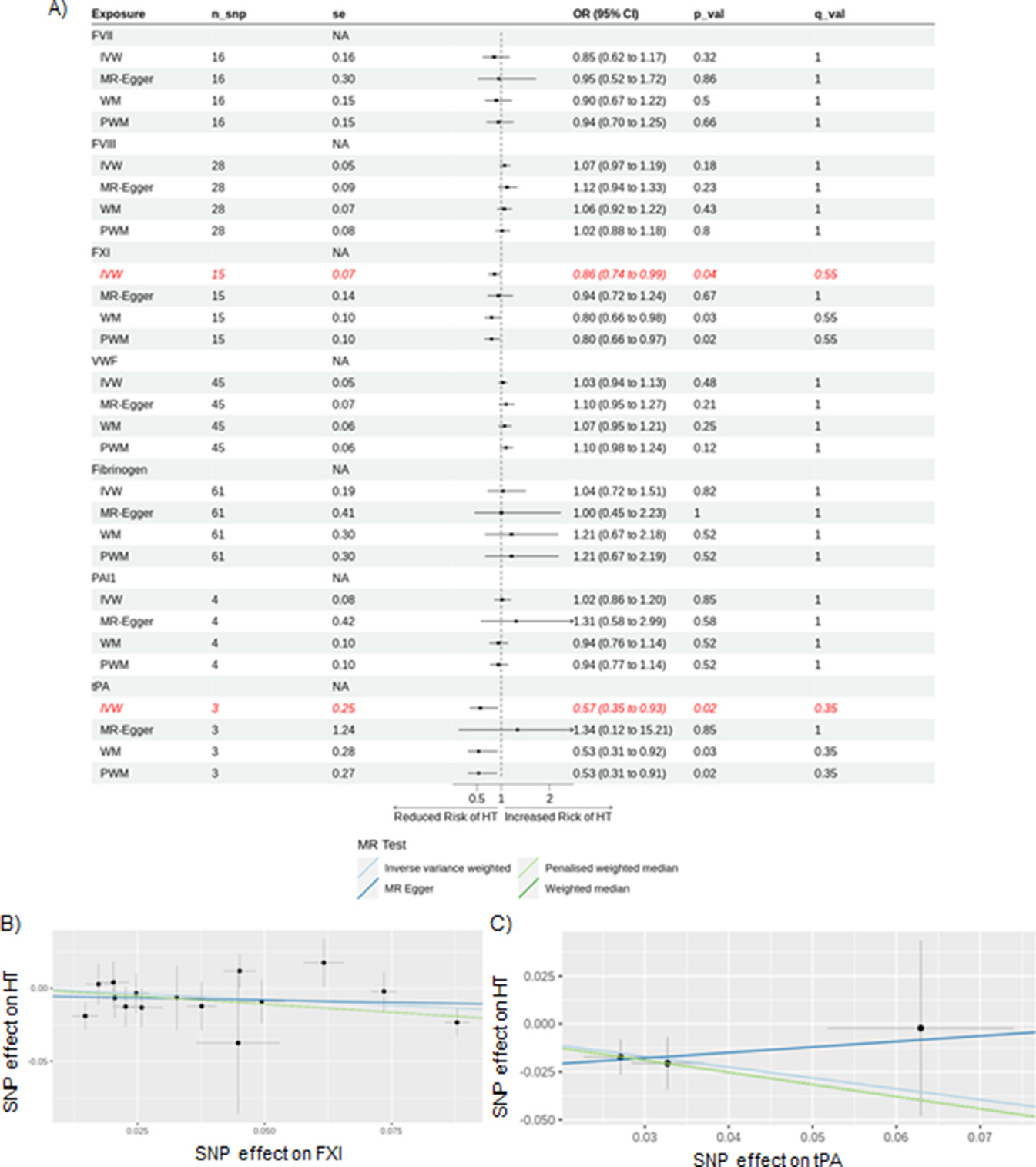

Supplement: Supplementary Figure S1 [file NIHMS1993459-supplement-Supplementary_Figure_S1.jpg]
